# Dihydrotanshinone as a Natural Product-Based CYP17A1 Lyase Inhibitor for Hyperandrogenic Disorders

**DOI:** 10.3390/biom16010144

**Published:** 2026-01-14

**Authors:** Kaige Li, Jibira Yakubu, Flemming Steen Jørgensen, Amit V. Pandey

**Affiliations:** 1 Pediatric Endocrinology, Diabetology and Metabolism, University Children’s Hospital, Inselspital, CH-3010 Bern, Switzerland; 2 Translational Hormone Research Program, Department of Biomedical Research, Faculty of Medicine, University of Bern, CH-3010 Bern, Switzerland; 3 Graduate School for Cellular and Biomedical Sciences, University of Bern, CH-3012 Bern, Switzerland; 4 Department of Drug Design and Pharmacology, University of Copenhagen, DK-2100 Copenhagen, Denmark

**Keywords:** CYP17A1, 17α-hydroxylase, CYP17A1 lyase, CYP21A2, steroidogenesis, hyperandrogenism, PCOS, selective inhibition

## Abstract

Selective inhibition of CYP17A1 17,20-lyase is critical for treating hyperandrogenic disorders without the cortisol-depleting side effects of non-selective drugs like abiraterone. We evaluated tanshinones from *Salvia miltiorrhiza* as potential selective inhibitors using biochemical assays and computational modeling. Dihydrotanshinone (DT) emerged as the superior candidate; at 10 µM, it inhibited 17,20-lyase activity by 56.6% while preserving >93% of 17α-hydroxylase activity. This yields a selectivity index of 8.67, drastically outperforming abiraterone (0.73). Furthermore, DT displayed minimal off-target inhibition of CYP21A2 (14.9%) compared to abiraterone (29.8%). Molecular modeling suggests DT’s efficacy arises from a unique, functionally disruptive binding pose rather than superior thermodynamic affinity. Consequently, DT is validated as a potent natural product lead. Its dual selectivity over 17α-hydroxylase and CYP21A2 establishes the tanshinone scaffold as a promising candidate for developing safer therapies that suppress androgens while sparing cortisol biosynthesis.

## 1. Introduction

**Figure 1:**
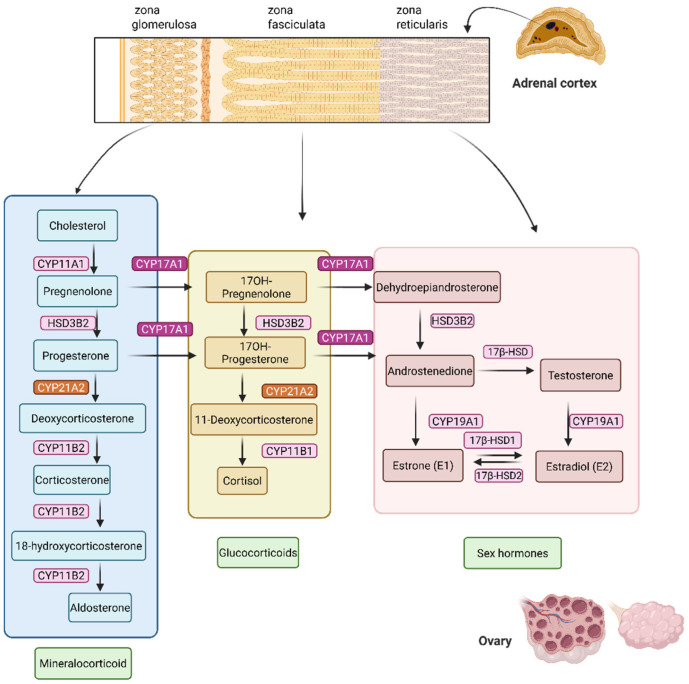
Steroid hormone biosynthesis in the ovary and adrenal cortex. Cholesterol serves as the precursor for all steroid hormones, undergoing enzymatic conversion through distinct pathways in the zona glomerulosa, zona fasciculata, and zona reticularis of the adrenal cortex, as well as in the ovary. Mineralocorticoid pathway (blue box) in the zona glomerulosa leads to aldosterone synthesis via the sequential action of CYP11A1, HSD3B2, CYP21A2, and CYP11B2. Glucocorticoid pathway (yellow box) in the zona fasciculata synthesizes cortisol through the enzymes CYP17A1, HSD3B2, CYP21A2, and CYP11B1. Specifically, CYP17A1 acts on pregnenolone or progesterone to add a 17α-hydroxyl group, producing 17α-hydroxypregnenolone or 17-hydroxyprogesterone, respectively. HSD3B2 converts 17α-hydroxypregnenolone to 17-hydroxyprogesterone (and pregnenolone to progesterone). CYP21A2 converts 17-hydroxyprogesterone to 11-deoxycortisol. CYP11B1 converts 11-deoxycortisol to cortisol. In the zona reticularis and ovarian theca/granulosa cells (pink box), androgens and estrogens are synthesized. CYP17A1 performs both 17α-hydroxylase and 17,20-lyase activities to produce dehydroepiandrosterone (DHEA) from 17α-hydroxypregnenolone. HSD3B2 converts DHEA to androstenedione, 17β-HSD converts androstenedione to testosterone. CYP19A1 (aromatase) converts androstenedione and testosterone into estrogens, such as estrone and estradiol. Enzyme names are shown in boxes next to each reaction step, with pathway products grouped by hormone class (mineralocorticoids, glucocorticoids, sex hormones).

Hyperandrogenism, the excessive production of androgens, is a primary driver of common and debilitating endocrine disorders, most notably polycystic ovary syndrome (PCOS) affecting 5–26% of women worldwide. PCOS is a leading cause of reproductive and metabolic dysfunction in women of reproductive age [1]. It is characterized by hyperandrogenism, menstrual irregularities, and polycystic ovarian morphology. Clinical manifestations include acne, alopecia, hirsutism, infertility, obesity, insulin resistance, and increased risk of cardiovascular disease, anxiety, and depression [2,3]. Similarly in congenital adrenal hyperplasia, elevated androgen levels are a constant challenge. Hyperandrogenism in PCOS is driven by dysregulated steroidogenesis, particularly overactivity of cytochrome P450 17A1 (CYP17A1) in ovarian theca cells. CYP17A1 is a dual-function enzyme with 17α-hydroxylase and 17,20-lyase activities, catalyzing key steps in the synthesis of dehydroepiandrosterone (DHEA) and androstenedione, precursors of testosterone [4,5,6] (Figure 1). Overexpression or increased activity of CYP17A1 due to changes in redox partner availability or protein phosphorylation leads to excessive androgen production, amplifying PCOS symptoms [7,8,9]. In contrast, CYP21A2 primarily regulates glucocorticoid and mineralocorticoid synthesis; partial deficiency as observed in congenital adrenal hyperplasia can also redirect precursors into androgen biosynthesis, further aggravating hyperandrogenemia [10,11]. First-line pharmacological treatments for PCOS, such as combined oral contraceptives (COCs) and metformin, reduce androgen levels indirectly [10,12,13]. COCs suppress gonadotropin release, lowering ovarian androgen production, while metformin improves insulin sensitivity, reducing hyperinsulinemia-driven androgen excess. However, these therapies are symptomatic, not curative, and are often associated with side effects such as abnormal uterine bleeding, thromboembolism, and gastrointestinal disturbances. Moreover, androgen levels often rebound after treatment discontinuation [14].

Direct CYP17A1 inhibition offers a more targeted approach to reducing androgen biosynthesis but the dual functionality of CYP17A1 presents a formidable therapeutic challenge [15,16,17,18,19,20]. Direct inhibition of CYP17A1 is a validated strategy for androgen suppression, exemplified by the drug abiraterone, which is used to treat castration-resistant prostate cancer [21]. However, abiraterone is a non-selective, “blunt instrument” inhibitor, potently blocking both the lyase and the essential hydroxylase activities of CYP17A1 [22,23] as well as inhibiting CYP21A2 [24,25]. This blockade of cortisol synthesis triggers a compensatory surge in adrenocorticotropic hormone (ACTH), leading to an accumulation of mineralocorticoid precursors and a subsequent syndrome of mineralocorticoid excess, characterized by hypertension, hypokalemia, and fluid retention. Consequently, abiraterone therapy necessitates co-administration with exogenous glucocorticoids to prevent adrenal insufficiency, a strategy that carries its own risks and is poorly suited for the long-term management of chronic, non-malignant conditions like PCOS and congenital adrenal hyperplasia. These limitations highlight the need for selective 17,20-lyase inhibitors that suppress androgens while sparing cortisol and mineralocorticoid production [26,27].

The search for novel chemical scaffolds capable of this precise selectivity has led to the investigation of natural products, a historically rich source of unique molecular architectures [28,29,30,31,32,33,34,35,36,37,38,39]. The root of *Salvia miltiorrhiza* (SM) (Danshen), a staple of traditional Chinese medicine, contains a class of bioactive diterpenoid quinones known as tanshinones [40,41,42,43,44]. These compounds, including tanshinone I (TAI), tanshinone IIA (TAII), and dihydrotanshinone 1 (DT), have demonstrated a wide range of pharmacological activities, and preliminary studies have suggested they may possess endocrine-modulating properties, making them compelling candidates for investigation as steroidogenesis inhibitors [44,45,46,47,48,49,50]. Their favorable tolerability profile compared to conventional pharmaceuticals positions them as promising candidates for long-term hormonal modulation [51,52].

This study was therefore designed to systematically investigate the inhibitory effects of a crude *SM* extract and its purified tanshinone constituents on the dual enzymatic activities of CYP17A1. By quantifying their impact on both 17α-hydroxylase and 17,20-lyase functions, as well as on the primary off-target steroidogenic enzyme CYP21A2, we aimed to determine their potential and selectivity as natural product-derived leads for the development of a new generation of safer, more effective therapies for hyperandrogenism.

## 2. Materials and Methods

### 2.1. Reagents and Materials

Dried roots of SM were purchased from Inner Mongolia Leihetang Company, Ordos, China. The material was naturally dried at room temperature, ground into a powder, and extracted with analytical-grade acetone. The powder (5 g) was soaked in acetone (100 mL) in an amber glass container and stored in the dark at room temperature for 24 h with intermittent shaking. The mixture was then filtered through filter paper. The residue was re-extracted once under the same conditions, and the filtrates were combined. The solvent was removed by passive evaporation (5–7 days) in a fume hood until the crude extract was dry. The extract was weighed to determine the extraction yield and stored in sealed amber vials at 4 °C (short-term) or −20 °C (long-term).

Abiraterone acetate was acquired from MedChemExpress through Lucerna Chem AG, (Lucerne, Switzerland). Purified tanshinone 1 (CAS# 568-73-0), tanshinone 11A (CAS# 568-72-9) and dihydrotanshinone 1 (CAS# 87205-99-0) were obtained from MedChemExpress (Monmouth Junction, NJ, USA). Radiolabeled substrates progesterone [4-^14^C] (specific activity: 55 mCi/mmol; concentration: 0.1 mCi/mL), and 17α-hydroxypregnenolone [21-^3^H] (specific activity: 15 Ci/mmol; concentration: 1 mCi/mL), were purchased from American Radiolabeled Chemicals Inc., (St. Louis, MO, USA). Non-radioactive compounds such as pregnenolone, progesterone, 17α-hydroxy pregnenolone, resazurin sodium salt, and dimethyl sulfoxide (DMSO) were procured from Sigma-Aldrich, St. Louis, MO, USA. Organic solvents were obtained from Carl Roth^®^ GmbH+ Co. KG, Karlsruhe, Germany, and activated charcoal from Merck AG, Darmstadt, Germany. Silica gel-coated aluminum-backed TLC plates were purchased from Macherey-Nagel, Oensingen, Switzerland. Tritium screens for autoradiography were provided by Fujifilm, Dielsdorf, Switzerland.

### 2.2. Cell Cultures

Human adrenocortical NCI-H295R cells (ATCC: CRL-2128) were purchased from ATCC and cultured in DMEM/Ham’s F-12 medium with L-glutamine and 15 mM HEPES (Thermo Fisher Scientific, Waltham, MA, USA), supplemented with 5% NU-Serum (Becton Dickinson, Franklin Lakes, NJ, USA), 0.1% insulin, transferrin, selenium (100 U/mL; Thermo Fisher Scientific, Waltham, MA, USA), and 1% penicillin (100 U/mL; Thermo Fisher Scientific, Waltham, MA, USA), and streptomycin (100 μg/mL; GIBCO, New York, NY, USA). RWPE-1 cells (ATCC: CRL-36O7) were cultured in keratinocyte serum-free media (K-SFM) supplemented with 0.05 mg/mL bovine pituitary extract and 5 ng/mL epidermal growth factor, and 1% penicillin (100 U/mL; Thermo Fisher Scientific, Waltham, MA, USA), and streptomycin (100 μg/mL; GIBCO). HEK-293T (ATCC: CRL-3216) cells were cultured in DMEM medium with L-glutamine and 15 mM HEPES-NaOH (Thermo Fisher Scientific, Waltham, MA, USA), supplemented with 10% fetal bovine serum (Thermo Fisher Scientific, Waltham, MA, USA), and 1% penicillin (100 U/mL; Thermo Fisher Scientific, Waltham, MA, USA), and streptomycin (100 μg/mL; GIBCO). Passage numbers remained below 30 as per standard procedures. All cell lines were validated and characterized before use to ensure their authenticity and reliability. Routine mycoplasma testing was conducted to confirm that the cells were free from contamination. Additionally, key functional and morphological characteristics were assessed to verify their alignment with previously reported profiles.

### 2.3. Alamar Blue Assay

The alamarBlue^®^ assay (Thermo Fisher Scientific, Waltham, MA USA) was conducted as previously described [15]. RWPE-1 cells, HEK-293 cells, and NCI-H295R cells were plated in 96-well plates at 10,000 cells per well and incubated overnight at 37 °C with 5% CO_2_. After 24 h, the medium was replaced with fresh medium containing the SM at a final concentration between 10–0.01 µg/mL and the pure chemicals from SM (TAI, TAII, and DT) at a final concentration between 10–0.01 µM. Cells were incubated for an additional 24 h. Cell viability was measured using the alamarBlue^®^ assay. Post-incubation, 0.05 mg/mL alamarBlue^®^ solution in phosphate-buffered saline (PBS) was added to each well. The plates were incubated in the dark at 37 °C with 5% CO_2_ for 4 h, and fluorescence was measured at an excitation wavelength of 540 nm and an emission wavelength of 590 nm. Cell viability was calculated relative to the control sample treated with DMSO. All experiments were conducted in triplicate [28,29].

### 2.4. CYP17A1 Assays

Assay of CYP17A1 17α-hydroxylase and 17,20-lyase activities were performed as described previously [15,25,53]. The 17α-hydroxylase activity was measured by conversion of progesterone to 17OH-progesterone, while production of DHEA from 17OH-pregnenolone was used for monitoring the 17,20-lyase activity of CYP17A1. In vitro CYP17A1 assays were performed using a liposome system consisting of human p450 oxidoreductase (POR) and CYP17A1 at a ratio of 3:4 (POR: CYP17A1). The final assay mixture consisted of proteins (37.5 nmol POR: 50 nmol CYP17A1), 10 mM MgCl_2_, 6 mM potassium acetate (KOAc), 0.05 mg/mL DLPC,1 mM reduced Glutathione in 50 mM HEPES-NaOH buffer (pH 7.4), and the reaction volume was 200 μL. The drugs were added to the final mixture and incubated for 1h before the catalytic reaction was initiated by the addition of NADPH to 1 mM final concentration. Radiolabeled [^14^C]-progesterone (10,000 cpm in 1 µM) was used as a tracer in each reaction. Steroids were extracted and separated by thin-layer chromatography (TLC) on silica gel (SIL G/UV254) TLC plates (Macherey-Nagel, Oensingen, Switzerland) as previously described [15,28].

In vitro CYP17A1 17,20-lyase activity assays were performed using POR, CYP17A1 and cytochrome b_5_ as described previously [15]. The final assay mixture consisted of proteins, 10 mM MgCl_2_, 6 mM KOAc, 0.05 mg/mL DLPC, 1 mM reduced glutathione in 50 mM HEPES-NaOH buffer (pH 7.4), and the reaction volume was 200 μL. The drugs were added to the final mixture and incubated for 1h before the catalytic reaction was initiated by the addition of NADPH to 1 mM final concentration. Radiolabeled [^3^H]-17OH-pregnenolone (50,000 cpm in 1 µM) was added as a tracer and the enzyme activity was measured by using the scintillation counter in the water release assay [28,29].

### 2.5. CYP21A2 Assays

Transient transfections were performed using the CYP21A2 WT vector as described previously [54]. HEK293 cells were seeded in six-well plates (3 × 10^5^ cells/well). After 24 h, the growth medium was replaced, and cells were transfected using Lipofectamine 2000 (Thermo Fisher Scientific, Bedford, MA, USA) with 12.5 µg of plasmid (plasmid concentration: 1068 µg/mL). Twenty-four hours after transfection, transfected cells were replated in 24-well plates (1.5 × 10^5^ cells/well) to ensure homogeneity of the cell population across the wells. Six hours after transfection, 2 mL of fresh medium was replaced, and the cells were cultured for 48 h. Cell growth was examined. If cells were growing well, SM, TAI, TAII, and DT were added at a concentration of 10 µg/mL for SM and 10 µM for TAI, TAII, and DT. DMSO was added to the negative control, and 10 µM abiraterone was added to the positive control, and the cells were incubated for 4 h. Functional assays were initiated by adding 1 µM unlabeled progesterone (using 10,000 cpm of [^14^C]-progesterone as a tracer). After incubation at 37 °C for 1 h, the culture medium and cells were harvested. Steroids were extracted from the culture medium with ethyl acetate and isooctane (1:1 volume ratio), dried, and dissolved in dichloromethane. Steroids were separated by thin-layer chromatography (TLC), placed on a phosphor screen, and visualized using a Typhoon PhosphorImager FLA-7000 (GE Healthcare Bio-Sciences AB, Uppsala, Sweden). Image intensity was measured and quantified using ImageQuant TL v8 (Cytiva, Marlborough, MA, USA). The CYP21A2 enzyme activity was expressed as relative steroid conversion. Steroids were quantified as a percentage of radioactivity incorporated into 11-deoxyprogesterone to the total radioactivity measured in the whole sample and compared between the WT and variants. Cells were collected with trypsin and washed with 1× PBS to quantify the amount of protein. Results were analyzed from three technical replicates. To ensure a similar amount of CYP21A2 in each reaction, a Western blot analysis was performed to normalize the enzyme activity with the relative CYP21A2 expression.

### 2.6. Western Blot

CYP21A2 expression was determined from total protein extracts. Cells were incubated with the previously described lysis buffer for 1 h and centrifuged at 10,000× *g* for 10 min at 4 °C [54]. The supernatant was collected, and total protein was determined using the Pierce Coomassie Plus (Bradford) assay kit (Thermo Fisher Scientific, Hanover Park, IL, USA). 50 μg of total protein was loaded onto an SDS-PAGE gel (GenScript, Piscataway, NJ, USA) and then transferred to a PVDF membrane as previously described. Two primary antibodies were used: a mouse DKY-Tag monoclonal antibody (GenScript, Catalog No. A00187) at a dilution of 1:1000 and a mouse anti-β-actin monoclonal antibody (Sigma Aldrich, St. Louis, MO, USA, Catalog No. SAB3500350) at a dilution of 1:1500. The secondary antibody, IRDye 800CW-conjugated donkey anti-mouse antibody (LI-COR, Lincoln, NE, USA, Catalog No. 926-32212), was used at a dilution of 1:15,000. Fluorescence signals were detected using an Odyssey SA infrared imaging system (LI-COR Bioscience Inc., Lincoln, NE, USA).

### 2.7. Computational Details

All calculations were done with the Schrödinger software system (Schrödinger Release 2025-2, Schrödinger, LLC, New York, NY, USA, 2025). Structures of the tanshinones were extracted from the PubChem database and subjected to the Ligand Preparation procedure in Maestro (v. 14.4.133) prior to the docking studies [55,56]. Protein structures were extracted from the Protein Data Bank and subjected to the Protein Preparation Procedure in Maestro [55,57]. Only the A-chain was used except for the 5IRQ structure, where both the A- and C-chains were used, since they contained different enantiomers of the ligand, *(R)-* and *(S)-*orteronel, respectively [58]. The GLIDE docking program (v2025-2 build 133) was used for the docking studies using the default setup. Receptor Grid Generation was defined by the ligand present in the CYP17A1 active site. The standard precision (SP) mode for scoring the poses was applied. For each compound up to 100 poses were collected and further refined by the default post-docking minimization available in GLIDE. The ten best scoring poses were visually inspected and subjected to MM-GBSA (Molecular Mechanics/Generalized Born Surface Area) energy calculation. The binding modes were described by a combination of the subjective terms “down/up” and “left/right” with reference to the orientation of the furane oxygen atom relative to the heme group. The MM-GBSA energy calculations were done with the Prime module (v2025-2 build 133) in Maestro. This method calculates both the free energy of binding and the individual energy contributions to the free energy. One representative of each binding mode of each compound was selected for molecular dynamics (MD) simulation. The MD simulations were performed with the Desmond program (v8.2.133) and comprised a default seven step equilibration procedure followed by a 50 ns production run at 300 K using the OPLS4 force field. For each simulation 50 frames were collected and analyzed. Structures were displayed with the Pymol program (v.3.1.5.1).

### 2.8. Data Analysis

Data analysis was conducted using RStudio (version 3.6.0+) and GraphPad Prism (GraphPad Software, Inc., San Diego, CA, USA) to ensure a thorough evaluation. Results are expressed as the mean ± standard deviation (SD) from three independent experiments to account for variability and reproducibility. One-way analysis of variance (ANOVA) was used to evaluate differences between treatment groups and controls, with post hoc analyses performed using Tukey’s Honest Significant Difference (HSD) test to identify specific differences. Significance was determined with thresholds of * *p* < 0.05 and ** *p* < 0.001. All tests were two-tailed, and assumptions of normality and variance homogeneity were checked prior to analysis to validate the ANOVA results.

## 3. Results

### 3.1. Computational Strategy

**Figure 2:**
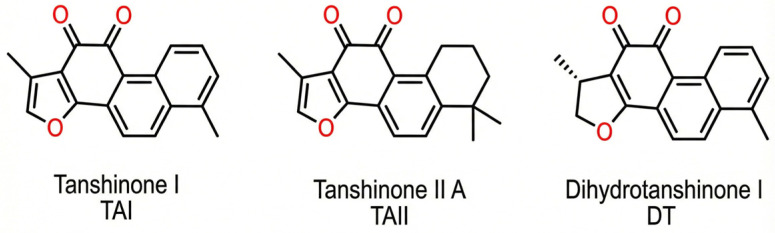
Structure of pure SM components tanshinone I, tanshinone IIa and dihydrotanshinone I.

Molecular docking and binding energy calculations of the ligands tanshinone IIA, dihydrotanshinone I, and tanshinone I (Figure 2) were employed to evaluate the potential binding affinity and orientation of selected tanshinone derivatives within the active site of human CYP17A1.

The computational workflow was designed to provide a comprehensive in silico assessment, moving from initial, broad-spectrum docking to more refined and energetically rigorous calculations. This process involved an exhaustive screen of all available CYP17A1 crystal structures to identify the most suitable model for a more detailed analysis. The A105L mutant bound to 17α-hydroxypregnenolone (PDB: 4NKZ, chain A) was selected for the subsequent docking studies due to its favorable and representative active site conformation for the compounds under investigation.

The final refinement of binding energy estimates was performed using MM-GBSA (Molecular Mechanics/Generalized Born Surface Area) calculations, a method that provides a more discriminative assessment of binding free energy by accounting for solvation effects and conformational entropy, which are often overlooked in simpler docking scores. MD simulations were performed on selected binding poses to explore the stability of the protein-ligand complex and flexibility of the ligand.

### 3.2. Molecular Docking and Binding Energy Analysis

**Figure 3:**
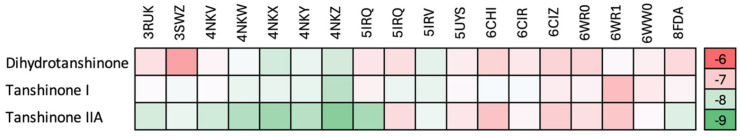
Heatmap showing the GLIDE docking scores for docking of the three tanshinones in the human CYP17A1 structures. Only the A-chain was used, except for 5IRQ, where both the A- and C-chains were used, since they contained different enantiomers of the ligand, *(R)-* and *(S)*-orteronel, respectively. The right column shows the scale in kcal/mol used in the heatmap.

Initially we performed a GLIDE based docking of the three tanshinones in all the human CYP17A1 structures available in the Protein Data Bank (Figure 3).

The docking score varies for the different structures reflecting that the active sites are slightly different in shape and charge distribution. The best scores are obtained for the CYP17A1 structures with the A105L mutation (4NKV, 4NKW, 4NKX, 4NKY and 4NKZ), which induces a minor conformational change in the active site supposed to make the structure more lyase like [59]. The subsequent studies were all done on the 4NKZ structure, as this was associated with the overall best score.

**Figure 4:**
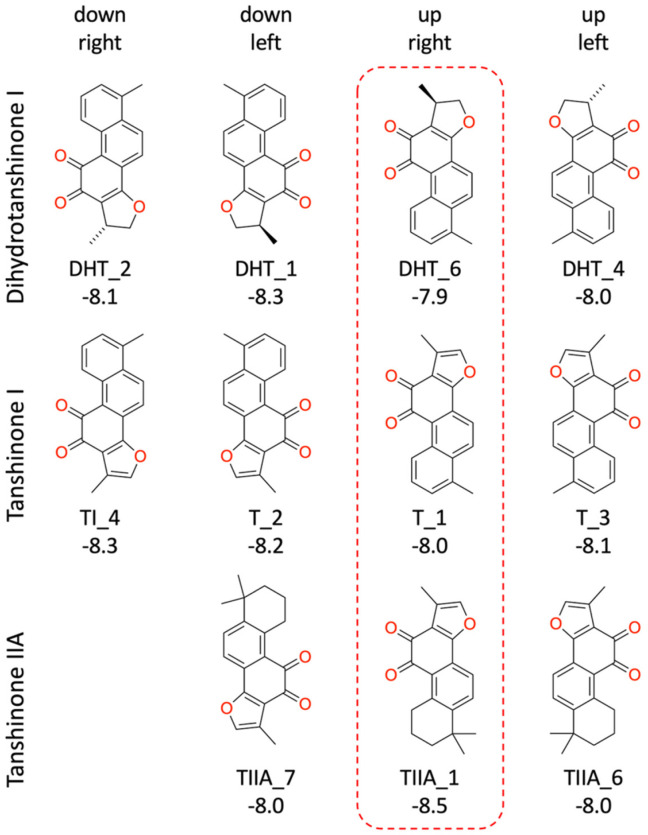
GLIDE binding modes for three tanshinones. The labels below each structure refer to the docking pose and the GLIDE docking score in kcal/mol. For data for all the GLIDE poses see Table 1.

**Figure 5:**
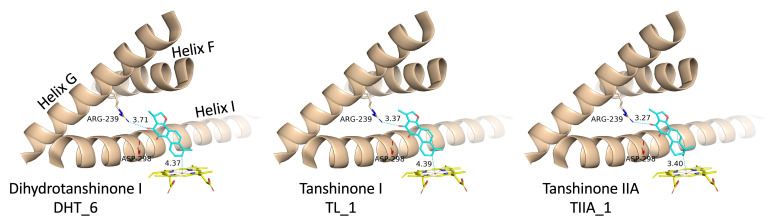
The “up–right” binding mode for the three tanshinones. For CYP17A1 Helix F, G and I are shown as a cartoon, Arg239, Asp298 and the heme group as type-colored stick models. The labels below the ligand names refer to the GLIDE poses (see Figure 4 and Table 1).

Inspection of the GLIDE poses revealed that there are no significant differences in the binding modes of the three tanshinones. The molecules are essentially planar and bind in four different ways with nearly identical shape. In the following descriptions, we refer to the four binding modes with a combination of the labels “up–down” and “left–right” reflecting their orientation to the heme group (Figure 4 and Figure 5, Table 1).

**Table 1:** Binding energies for the GLIDE docking poses. See text for details on the different energy terms. Binding mode refers to the binding modes described in Figure 4. Each energy column is color coded continuously with lowest energy value green and highest energy value red. Poses in yellow boxes were selected for MD simulations. All energies are kcal/mol.

Compound	Pose	Docking Score	GLIDE Emodel	MMGBSA dG Bind	Binding Mode
Dihydrotanshinone I	1	−8.2	−62.2	−39.5	down–left
	2	−8.1	−62.1	−30.6	down–right
	3	−8.1	−62.2	−41.0	down–left
	4	−8.0	−59.5	−35.2	up–left
	5	−8.0	−60.6	−39.4	down–left
	6	−7.9	−58.5	−39.3	up–right
	7	−7.9	−60.6	−31.5	down–right
	8	−7.8	−59.9	−34.2	up–left
	9	−7.7	−58.4	−39.3	up–right
	10	−6.0	−30.3	−6.7	up–right
Tanshinone I	1	−8.3	−62.7	−44.4	up–right
	2	−8.2	−64.6	−40.8	down–left
	3	−8.1	−61.6	−36.4	up–left
	4	−8.0	−62.6	−40.2	down–right
	5	−8.0	−61.4	−44.4	up–right
	6	−7.9	−58.5	−42.4	down–left
	7	−7.8	−56.7	−39.1	down–left
	8	−7.6	−53.8	−37.9	up–left
	9	−7.0	−50.2	−44.3	up–right
	10	−5.9	−25.7	2.1	skewed
Tanshinone IIA	1	−8.5	−62.1	−34.9	up–right
	2	−8.3	−59.8	−41.6	up–right
	3	−8.2	−59.7	−26.9	up–left
	4	−8.1	−59.8	−41.8	up–right
	5	−8.1	−59.9	−47.1	up–right
	6	−8.0	−58.8	−29.6	up–left
	7	−7.9	−56.7	−31.6	down–left
	8	−7.8	−56.7	−26.2	down–left
	9	−7.8	−57.3	−32.1	up–left
	10	−7.6	−50.9	−20.1	down–left

The “up–right” binding mode, which represents the best scoring pose for dihydrotanshinone I and tanshinone I, is especially interesting, because it is the only binding mode with a direct contact to the protein, a hydrogen-bond to Arg239 (Figure 5).

Docking programs are generally better to predict correct binding modes than binding energies, because the former primarily is controlled by specific interactions like hydrogen-bonding and charge-charge interactions whereas binding energies often depend on more diffuse interactions like non-polar interactions and hydrophobic effects like desolvation and solvation [60].

In Table 1 we have collected the binding scores/energies for the different binding modes. If we use the approach recommended by Schrodinger, the lowest E model energy corresponds to the preferred binding mode for each ligand, whereas the docking score should be used to compare binding of different ligands (https://my.schrodinger.com/support/article/1027 accessed on 1 October 2025). Nevertheless, the differences in docking scores are too small to be significant and, accordingly, we have subjected the GLIDE poses to a more rigorous energy determination method. The MM-GBSA method is a molecular mechanics-based (MM) method for calculation of binding energies combined with an implicit continuum treatment of solvation by a generalized Born (GB) and solvent area (SA) based approach [61]. The MM-GBSA method is often used for post-evaluation of docking scores to achieve better binding energies [62,63].

MM-GBSA calculations, which estimate the free energy of binding, predicted that TAI (−44.32 kcal/mol) and TAII (−41.73 kcal/mol) were thermodynamically more favorable binders than DT (−40.95 kcal/mol) (Table 1). This is in direct opposition to our experimental data, where DT is the most potent functional inhibitor. This apparent discrepancy is a critical finding, suggesting that static binding affinity alone does not dictate inhibitory efficacy. Instead, the distinct binding pose adopted by DT, though potentially less stable, appears to be more functionally disruptive to the lyase catalytic cycle. This highlights the limitation of using thermodynamic energy scores as the sole predictor of biological activity and points toward the paramount importance of specific inhibitor–enzyme geometry for functional outcomes [64,65].

### 3.3. Molecular Dynamics Simulations

**Figure 6:**
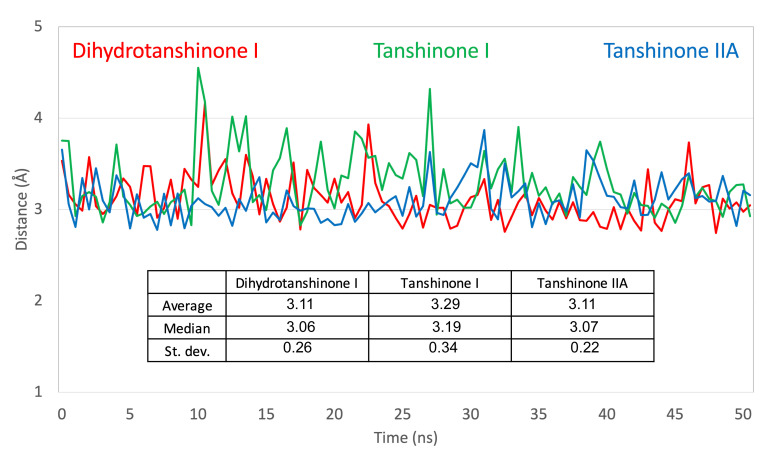
Variation in the hydrogen bond between Arg239 and tanshinone ligands. Dynamic behavior of the key Arg239-ligand hydrogen bond in the ‘up–right’ binding pose during 50 ns MD simulations. The plot displays the variation of hydrogen bond distances (in Ångstroms) between the guanidinium group of Arg239 and one of the proximal carbonyl oxygen of each tanshinone ligand. The inset table provides key statistics, including the mean distance and standard deviation.

**Figure 7:**
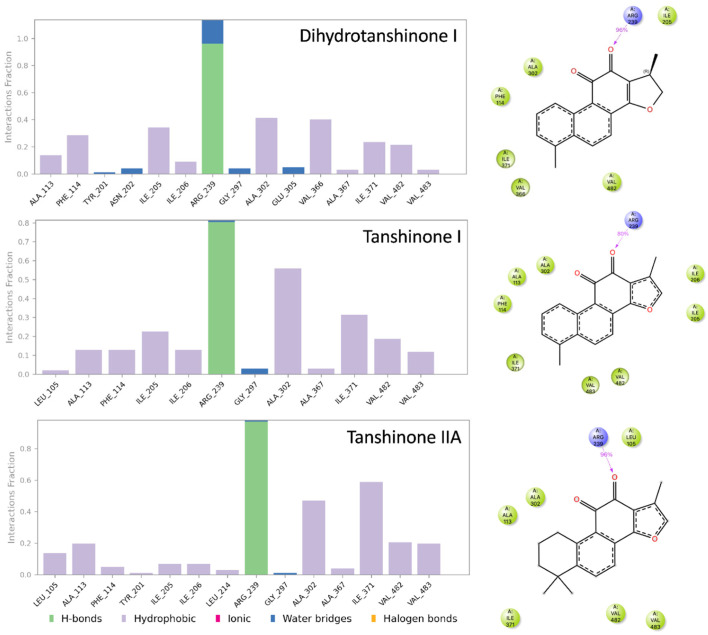
Simulation interaction diagrams. The bar diagrams (**left**) show the interactions between the protein CYP17A1 and the ligands during the MD simulation. Key to type of interactions shown below bar diagrams. The structures (**right**) display the intramolecular interactions present in more than 30% of the frames sampled during the MD simulations. Light green and dark blue symbols refer to hydrophobic and hydrogen-bonding interactions, respectively.

To further characterize the binding of the tanshinones to CYP17A1 we subjected one representative (with the best docking score) of each of the eleven binding modes shown in Figure 4 to short MD simulations. In all eleven MD simulations the systems were stable and the ligand remained in the active site. Nevertheless, the complexes with the “up–right” binding mode had significantly smaller RMSD values for the protein Ca atoms. The ligands, the tanshinones, also displayed lower RMSD values relative to the protein than observed for the other binding modes. Thus, we conclude, that the “up–right” binding mode with the hydrogen-bond from Arg239 to one of the ligand carbonyl oxygens has a stabilizing effect on the complexes. The Arg239-ligand hydrogen-bond fluctuate, but we see a preference for a slightly shorter hydrogen bond and a slightly smaller variation for dihydrotanshinone I and tanshinone IIA than for tanshinone I (Figure 6 and Figure 7), suggesting a stronger hydrogen-bond for these two ligands. This interaction is hypothesized to be a critical anchor point that orients the planar tanshinone scaffold within the active site, contributing to the stability and potentially the inhibitory activity of the compounds.

### 3.4. Cell Lines and Cytotoxicity Assays

#### 3.4.1. Cytotoxicity of Tanshinones in Adrenal, Kidney, and Prostate Cell Lines

**Figure 8:**
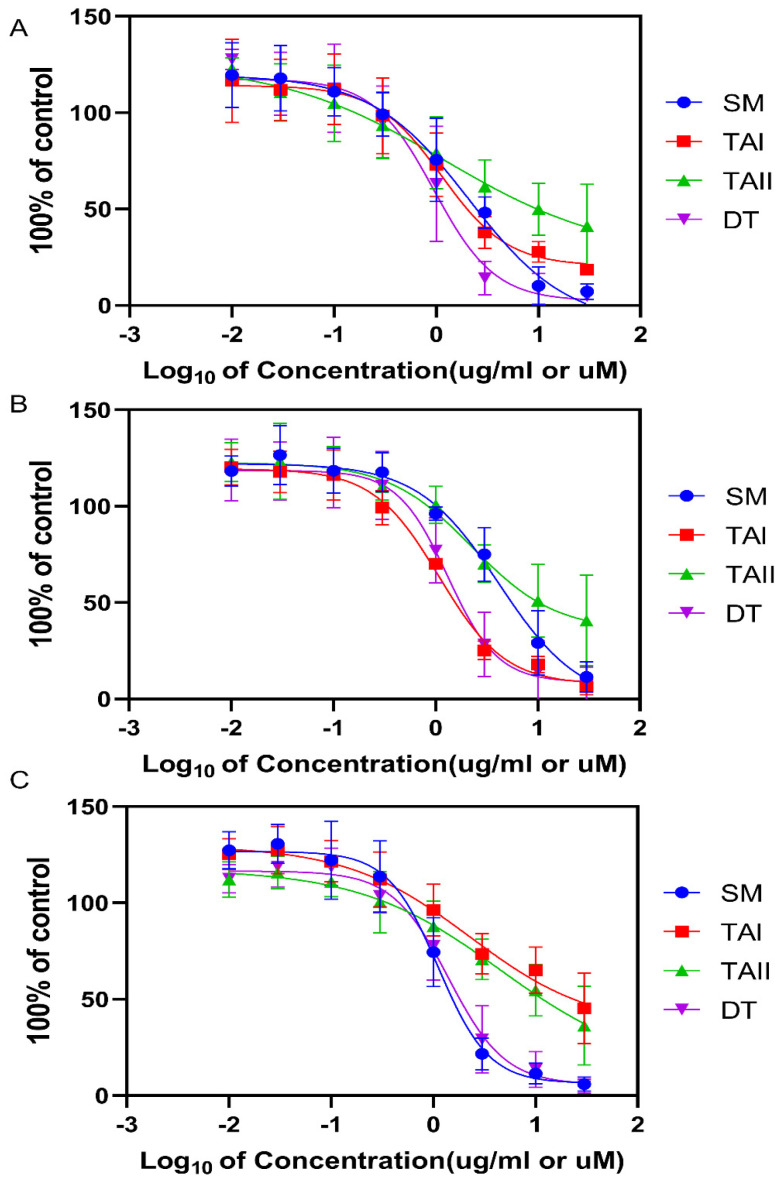
Cytotoxicity of *SM* extract and purified tanshinones in human cell lines. Dose–response curves show the effect of increasing concentrations of SM extract (µg/mL), TAI (µM), TAII (µM), and DT (µM) on the viability of (**A**) NCI-H295R adrenocortical, (**B**) HEK-293T kidney, and (**C**) RWPE-1 prostate epithelial cells after 24-h incubation. Cell viability was determined using the alamarBlue^®^ assay and is expressed as a percentage of the vehicle (DMSO) control. Data points represent the mean ± SD of three independent experiments. The IC_50_ values derived from these curves are summarized in Table 2.

To establish a therapeutic window and determine appropriate concentrations for subsequent enzyme inhibition assays, the cytotoxic effects of the SM extract and its purified components were assessed across three distinct human cell lines: NCI-H295R (adrenocortical carcinoma), HEK-293T (embryonic kidney), and RWPE-1 (normal prostate epithelium). As summarized in Table 2 and Figure 8, dihydrotanshinone (DT) exhibited the most consistent cytotoxic effects across all three cell lines, with half-maximal inhibitory concentrations (IC_50_) ranging from 0.95 μM to 1.40 μM. The other pure compounds, TAI and TAII, also induced cell death in the low single-digit micromolar range. However, the data for TAI and TAII showed greater variability between cell lines and larger experimental variance, particularly in the RWPE-1 cell line (e.g., TAII IC_50_ 4.50 μM), which precludes a precise comparative ranking of their potency. The crude SM extract was cytotoxic in the low μg/mL range, with the highest potency observed in the RWPE-1 cell line (IC_50_ 1.12 μg/mL). Based on these findings, a non-toxic concentration of 10 μM for the pure compounds and 10 μg/mL for the SM extract was selected for all subsequent enzyme activity assays.

#### 3.4.2. In Silico Assessment of Physicochemical Properties and Pharmacokinetic Profile

To build a more comprehensive preclinical profile and anticipate potential developmental challenges, a thorough in silico analysis of the absorption, distribution, metabolism, and excretion (ADME) properties of the lead tanshinones was performed using the SwissADME toolkit [66]. We compared the properties of tanshinone I (TAI), tanshinone IIA (TAII), and the most potent and selective inhibitor, dihydrotanshinone I (DT). The analysis of fundamental physicochemical properties reveals that all three compounds are well within the ranges defined by established oral drug-likeness filters (Table 3). All compounds possess molecular weights under 300 g/mol, a favorable consensus Log P (lipophilicity) between 3.0 and 3.8, and, critically, zero violations of Lipinski’s Rule of Five, suggesting a high potential for oral bioavailability. A notable distinction emerged in the overall bioavailability score, a composite metric that integrates multiple physicochemical parameters. DT is predicted to have a superior bioavailability score of 0.85, compared to 0.55 for both TAI and TAII. This advantage for DT appears to be driven by its slightly lower lipophilicity (Consensus Log P = 3.03) and significantly higher predicted aqueous solubility (ESOL Class: Soluble) relative to the other two analogs. This predicted enhancement in oral bioavailability represents a significant advantage, as it can directly translate to improved dosing regimens, better therapeutic efficacy, and enhanced patient compliance, further strengthening the rationale for prioritizing DT as the lead scaffold.

Predicted pharmacokinetic behaviors indicate that all three compounds are likely to exhibit high gastrointestinal (GI) absorption and are capable of permeating the blood–brain barrier (BBB). While high GI absorption is desirable, the predicted BBB permeability raises the possibility of central nervous system (CNS) exposure, which could lead to off-target neurological effects. This is a critical parameter that would require careful monitoring in subsequent in vivo preclinical safety studies. A key predicted differentiator was found in their interaction with the P-glycoprotein (P-gp) efflux pump, a major mechanism of drug resistance. While TAI and DT are not predicted to be P-gp substrates, TAII is, which could limit its effective intracellular and tissue concentrations and potentially compromise its efficacy.

The in silico models predict that the tanshinone scaffold may inhibit other CYP isoforms. All three compounds are predicted to be inhibitors of CYP1A2 and CYP2C19. Furthermore, TAII and DT are also predicted to inhibit CYP2C9, CYP2D6, and CYP3A4. This predicted broad-spectrum CYP inhibition represents a major developmental red flag. The five enzymes predicted to be inhibited by DT (CYP1A2, CYP2C19, CYP2C9, CYP2D6, and CYP3A4) are collectively responsible for the metabolism of over 90% of all clinically prescribed drugs. The target patient populations for a CYP17A1-lyase inhibitor, such as individuals with PCOS or congenital adrenal hyperplasia, frequently have comorbidities and are often on multiple medications (e.g., metformin, oral contraceptives, statins, glucocorticoids), many of which are substrates for these CYP enzymes. Co-administration of a potent CYP inhibitor like DT could elevate the plasma concentrations of these concomitant medications, leading to toxicity. Finally, structural analysis using medicinal chemistry filters identified potential intrinsic liabilities. All three compounds registered two PAINS (Pan-Assay Interference Compounds) alerts, specifically for the “imine_one_A_quinone_D” substructure, and one Brenk alert for a “diketo_group”. These alerts flag chemical motifs that are known to be potentially reactive, prone to non-specific binding, and can interfere with assay readouts. The presence of these reactive moieties may mechanistically underlie not only the observed cytotoxicity but also some of the predicted toxicity endpoints.

#### 3.4.3. Predictive Toxicological Assessment

To identify potential safety liabilities and guide future optimization efforts, a comprehensive in silico toxicological assessment was conducted using the ProTox-3.0 server (Table 4) [67]. At the organ level, the predictions were generally favorable for several critical systems. None of the three tanshinones were predicted to be hepatotoxic, neurotoxic, nephrotoxic, or cardiotoxic. However, all three compounds were predicted to be active for respiratory toxicity, with moderate-confidence probabilities ranging from 0.56 for TAII to 0.66 for DT. More significant toxicological flags were raised for systemic toxicity endpoints, where clear differences between the analogs emerged. While TAI and TAII were predicted to be inactive, DT was predicted to be carcinogenic with borderline confidence (Prob. 0.50). More alarmingly, DT was also predicted to be immunotoxic with very high confidence (Prob. 0.98). This high-probability prediction for immunotoxicity is a concerning finding from the in silico safety assessment. Immunotoxicity can manifest as either immunosuppression or inappropriate immune activation (hypersensitivity), and it is a frequent cause of attrition for drug candidates in later stages of development. The exceptionally high confidence score of 0.98 suggests that the predictive model identified strong structural precedents for this liability within its training data. A plausible mechanistic basis for this prediction can be formed by linking it to the structural alerts identified previously. The quinone-type substructure flagged by the PAINS analysis is a known structural class of haptens, small, reactive molecules that can covalently bind to endogenous proteins. This creates neoantigens that are subsequently recognized as foreign by the immune system, triggering a drug-specific immune response.

Analysis of molecular-level interactions provided further mechanistic hypotheses for potential off-target effects. All three compounds were predicted to be active at the GABA receptor, and DT was additionally predicted to inhibit acetylcholinesterase (AChE), suggesting potential for neurological side effects. A particularly complex interaction was predicted with the Pregnane X Receptor (PXR), a key nuclear receptor that governs the transcriptional regulation of many drug-metabolizing enzymes and transporters. Both TAII and DT were predicted to be PXR activators. This creates a complicated regulatory scenario when viewed in conjunction with the ADME predictions. PXR activation leads to the transcriptional induction of the *CYP3A4* gene, increasing the synthesis of CYP3A4 protein. However, the SwissADME data (Table 3) concurrently predicts that these same compounds act as direct inhibitors of CYP3A4 activity. This dual activity, enzyme induction at the genetic level and direct inhibition at the protein level, can lead to highly complex and unpredictable drug–drug interaction profiles in vivo. The net effect could change over time, with initial inhibition of drug metabolism potentially followed by a return to normal or even accelerated metabolism as more enzyme is produced. This complex interaction profile further elevates the DDI risk and reinforces the conclusion that the tanshinone scaffold requires significant medicinal chemistry optimization to be clinically viable.

### 3.5. Tanshinones Selectively Inhibit CYP17A1 17,20-Lyase Activity While Sparing 17α-Hydroxylase Activity

**Figure 9:**
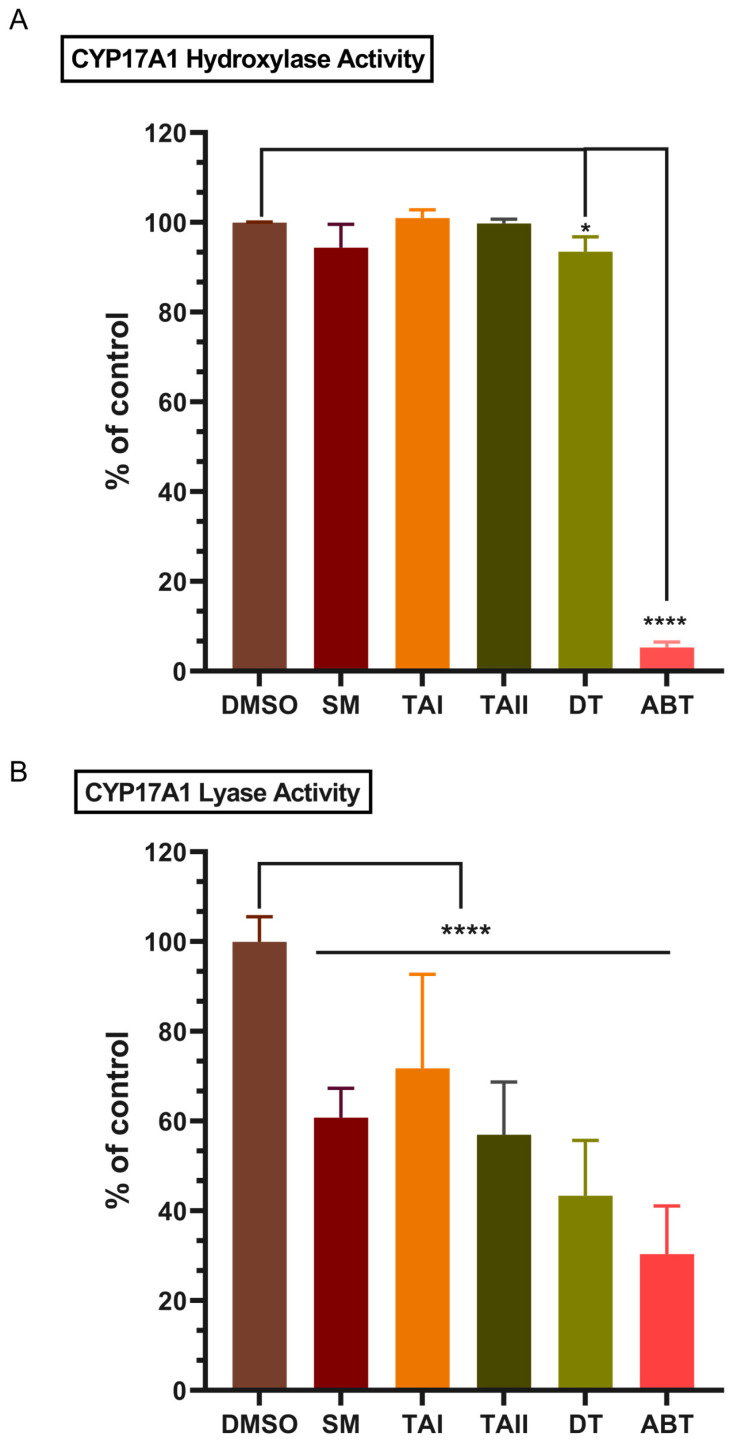
Selective inhibition of CYP17A1 17,20-lyase activity by tanshinones. The residual enzymatic activity of (**A**) CYP17A1 17α-hydroxylase and (**B**) CYP17A1 17,20-lyase following incubation with vehicle (DMSO), abiraterone (ABT, 10 µM), *Salvia miltiorrhiza* extract (SM, 10 µg/mL), TAI (10 µM), TAII (10 µM), and DT (10 µM). Activities were measured in a reconstituted microsomal system and are expressed as a percentage of the DMSO control. Data represent the mean ± SD from three independent experiments. Statistical significance was determined by one-way ANOVA with Tukey’s post hoc test. * *p* < 0.05, **** *p* < 0.001. The data demonstrates that DT and SM selectively inhibit the lyase reaction while sparing the hydroxylase function, unlike the non-selective inhibitor abiraterone.

The primary objective was to assess the ability of tanshinones to selectively inhibit the androgen-producing 17,20-lyase function of CYP17A1 while sparing its 17α-hydroxylase function. In a reconstituted microsomal system, none of the tested tanshinones or the SM extract significantly inhibited 17α-hydroxylase activity at concentrations of 10 µM or 10 µg/mL, respectively. Residual hydroxylase activity remained above 93% for all natural compounds, in stark contrast to the non-selective inhibitor abiraterone, which ablated this activity to just 5.3% of the control (Figure 9A).

These data provide the first line of evidence for the preferential inhibition of the lyase reaction by the tanshinone scaffold. Conversely, the compounds demonstrated clear inhibitory effects on 17,20-lyase activity (Figure 9B). Dihydrotanshinone (DT) emerged as the most potent inhibitor among the natural compounds, reducing lyase activity to 43% of the control. The crude SM extract also showed significant inhibition (61% residual activity), followed by TAII (57%) and TAI (72%). Abiraterone, as expected, was the most potent inhibitor overall, with a residual activity of 30%.

### 3.6. Tanshinones Exhibit a Favorable Safety Profile with Minimal Inhibition of CYP21A2

**Figure 10:**
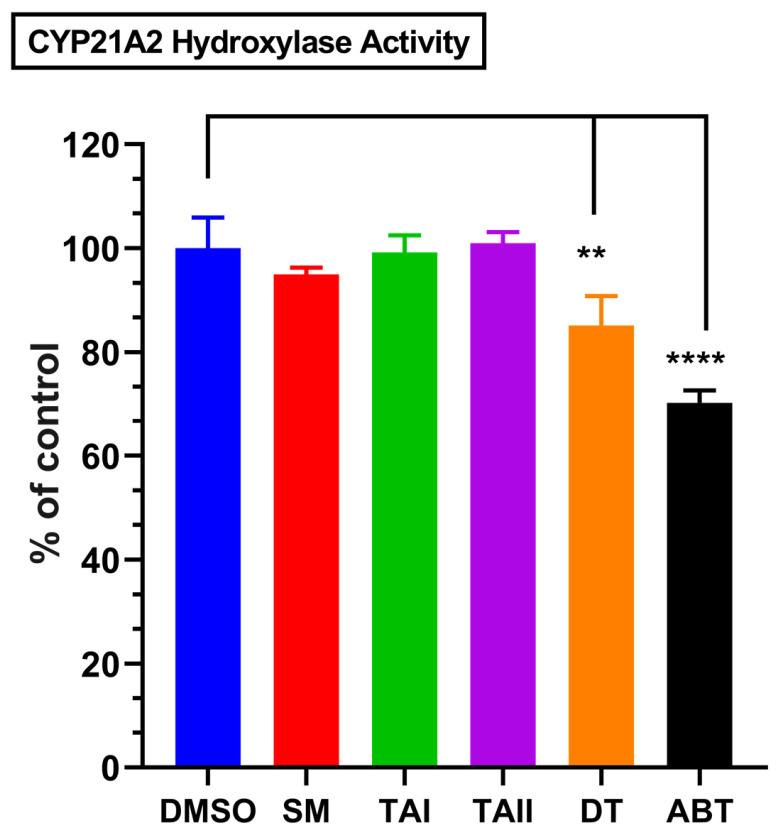
Minimal inhibition of off-target CYP21A2 by tanshinones. The bar chart displays the residual enzymatic activity of CYP21A2 following incubation with vehicle (DMSO), abiraterone (ABT, 10 µM), *Salvia miltiorrhiza* extract (SM, 10 µg/mL), TAI (10 µM), TAII (10 µM), and DT (10 µM). Activity was measured in transiently transfected HEK-293T cells and is expressed as a percentage of the DMSO control. Data represent the mean ± SD from three independent experiments. Statistical significance was determined by one-way ANOVA with Tukey’s post hoc test. ** *p* < 0.01, **** *p* < 0.001. The results show that DT exhibits only modest inhibition of CYP21A2, while other natural compounds have negligible effects, contrasting with the significant inhibition caused by abiraterone.

A critical liability of non-selective CYP17A1 inhibitors like abiraterone is the concurrent inhibition of CYP21A2 (21-hydroxylase), an enzyme essential for cortisol and aldosterone synthesis. Such off-target inhibition disrupts the glucocorticoid pathway, leading to clinically significant side effects. To assess the potential for this liability, the inhibitory effects of the compounds on CYP21A2 activity were evaluated in HEK-293T cells transiently expressing the enzyme. A critical liability of abiraterone is its off-target inhibition of CYP21A2. When tested in a cell-based assay, the SM extract, TAI, and TAII showed no meaningful inhibition of CYP21A2. DT exhibited only modest inhibition (85% residual activity), a profile that contrasts sharply with abiraterone, which significantly reduced CYP21A2 activity to 70% of the control (Figure 10). To quantify the degree of selectivity, we calculated selectivity indices based on the observed inhibition percentages (Table 5). This analysis revealed that DT possesses an 8.67-fold selectivity for the lyase over the hydroxylase reaction, a stark contrast to the non-selective profile of abiraterone (index = 0.73). Furthermore, DT demonstrated a favorable therapeutic index against the primary off-target, inhibiting the lyase reaction 3.8-fold more potently than CYP21A2. This finding suggests that tanshinone-based inhibitors may avoid the downstream metabolic consequences that complicate abiraterone therapy.

### 3.7. Inhibition of CYP21A2 Is Mediated by Direct Catalytic Interference, Not Altered Protein Expression

**Figure 11:**
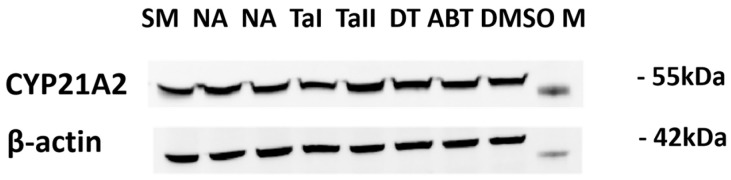
Effect of tanshinones on CYP21A2 Protein Expression. Representative Western blot of CYP21A2 protein levels in transiently transfected HEK-293T cells treated for 4 h with vehicle (DMSO), abiraterone (ABT), SM, TAI, TAII, or DT. β-actin was used as a loading control and densitometric quantification of CYP21A2 protein bands was performed by normalization to β-actin. Data is expressed relative to DMSO control. The results show that the modest inhibition of CYP21A2 activity by DT is not associated with a decrease in protein expression, indicating a direct effect on enzyme catalysis rather than protein stability or synthesis. The original Western blot images in Supplementary Materials also include two extra compounds Marjoram and Chinese dodder (shown as NA), which are not part of the current study and were loaded as part of a larger set.

To investigate whether the observed effects on CYP21A2 activity were attributable to changes in protein levels, a Western blot analysis was performed on lysates from the treated HEK-293T cells. Interestingly, the changes in CYP21A2 protein expression did not correlate with the enzymatic activity data (Figure 11). For instance, treatment with DT and TAII led to a slight increase in CYP21A2 protein levels (normalized densitometry values of 1.15 and 1.30, respectively) compared to the DMSO control (1.07). This occurred despite DT causing modest enzymatic inhibition and TAII having no effect on activity.

This discordance suggests that the observed enzymatic inhibition by DT is a direct effect on the catalytic function of the CYP21A2 enzyme itself, rather than a consequence of reduced protein expression. The modest upregulation of CYP21A2 protein in response to some treatments may reflect a secondary cellular feedback or stress response, where the cell attempts to compensate for perceived pathway disruption by increasing enzyme synthesis. This rules out transcriptional or translational downregulation as the primary mechanism of action for the observed effects on CYP21A2.

## 4. Discussion

This study provides compelling evidence that dihydrotanshinone (DT) and the crude extract of SM function as novel, natural product-derived selective inhibitors of the 17,20-lyase activity of CYP17A1. The central finding is that these compounds, particularly DT, potently inhibit the final androgen-producing step in the steroidogenic cascade while demonstrating markedly reduced activity against both the 17α-hydroxylase function of CYP17A1 and the critical off-target enzyme, CYP21A2. This dual selectivity addresses a major challenge in endocrine pharmacology and represents a significant advancement toward a safer therapeutic profile for managing hyperandrogenic disorders like PCOS and certain forms of prostate cancer.

The therapeutic imperative for such selectivity is best understood in the context of the current clinical standard, abiraterone. Abiraterone is a potent and irreversible inhibitor of both CYP17A1 activities. By blocking the 17α-hydroxylase reaction, it shuts down the production of cortisol. The resulting hypocortisolism triggers a compensatory surge in pituitary adrenocorticotropic hormone (ACTH), which in turn overstimulates the upstream steps of the adrenal cascade. This leads to an accumulation of mineralocorticoid precursors and the development of a mineralocorticoid excess syndrome, characterized by hypertension, hypokalemia, and fluid retention. To mitigate these serious side effects, abiraterone must be co-administered with a glucocorticoid like prednisone, which creates its own set of long-term therapeutic burdens. The compounds identified in this study, by sparing both 17α-hydroxylase and CYP21A2, have the potential to uncouple androgen suppression from cortisol disruption. This profile could obviate the need for glucocorticoid co-therapy, a profound clinical advantage, especially for chronic, non-malignant conditions like congenital adrenal hyperplasia and PCOS where long-term safety is paramount.

The structural basis for this observed selectivity can be rationalized through molecular modeling, although such in silico approaches must be interpreted with caution as hypothesis-generating tools rather than definitive proof [68,69]. Structurally, CYP17A1’s dual catalytic functions mediated by separate active sites for hydroxylase and lyase enable the design of selective inhibitors [70]. SM and DT likely bind preferentially to the lyase site or induce conformational changes that selectively impair lyase function without affecting hydroxylase catalysis. This hypothesis is supported by recent structure–function studies and molecular docking analyses. This complexity of conformational changes upon substrate binding or due to interaction with cytochrome b_5_, offers multiple avenues through which a small molecule could selectively interfere with only the second catalytic step. The docking models provide a structural hypothesis for the selectivity of DT that can now be tested experimentally through techniques like site-directed mutagenesis.

The primary finding of this study is the potent and selective inhibition of CYP17A1 17,20-lyase activity by DT. DT reduced the lyase activity to 43% of control levels while having negligible effects on 17α-hydroxylase function, which remained above 93% of control. This exceptional selectivity is quantitatively supported by the calculated selectivity index of 8.67 for DT, which dramatically surpasses the index of 0.73 for the non-selective CYP17A1 inhibitor abiraterone. A central mechanistic insight from our study emerges from the apparent paradox between the computational binding energy predictions and the experimental enzyme inhibition data. While MM-GBSA calculations suggested DT was the weakest binder of the three tanshinones, it was experimentally the most potent inhibitor. This is likely not a model failure but rather a reflection of the complex biology of CYP17A1. The dual activities of this enzyme are thought to depend on subtle conformational states and allosteric regulation by the redox partner cytochrome b_5_, which is essential for lyase but not hydroxylase activity. The unique binding pose adopted by DT, while perhaps not the most thermodynamically stable, may be optimally positioned to disrupt the specific protein dynamics or substrate reorientation required for the C17-C20 bond cleavage, while not interfering with the initial hydroxylation step. The simulations also revealed a preference for a shorter, less variable hydrogen bond for dihydrotanshinone I and tanshinone IIA compared to tanshinone I, suggesting a more persistent and stable interaction that may contribute to their inhibitory mechanism. This ‘function-over-affinity’ hypothesis provides a powerful framework for future structure-based drug design, suggesting that optimizing for a specific inhibitory geometry, rather than raw binding energy, will be the key to enhancing potency and selectivity.

This selective inhibition profile of the tanshinones, particularly DT, represents a major pharmacological advancement over the current clinical standard, abiraterone. Abiraterone’s blunt instrument approach, which potently inhibits both the 17α-hydroxylase and 17,20-lyase activities, necessitates co-administration with exogenous glucocorticoids to prevent the serious side effects of hypocortisolism and mineralocorticoid excess. The capacity of DT to spare the 17α-hydroxylase function, thereby maintaining cortisol production, could obviate the need for glucocorticoid co-therapy, a profound clinical advantage for the long-term management of chronic, non-malignant conditions like polycystic ovary syndrome (PCOS) and congenital adrenal hyperplasia.

Furthermore, the study provides a deeper understanding of the compound’s mechanism of action by analyzing its effects on the off-target enzyme, CYP21A2. The data shows that while abiraterone significantly inhibits CYP21A2 activity, DT causes only a modest inhibition. The Western blot analysis provides a crucial piece of mechanistic evidence: the observed inhibition of CYP21A2 activity occurs in the absence of a decrease in protein expression. In fact, treatment with DT and TAII led to a slight increase in CYP21A2 protein levels. This finding definitively rules out a mechanism of inhibition based on transcriptional or translational downregulation and strongly supports a direct, catalytic inhibition of the enzyme. The slight upregulation of the protein may represent a compensatory cellular feedback loop, where the cell attempts to increase enzyme synthesis to overcome the direct catalytic block.

The inhibitory action of SM extract on CYP17A1 observed in our in vitro assays appears to conflict with some in vivo studies reporting that *SM* can restore or even enhance steroidogenesis and testosterone levels. This discrepancy is not uncommon with natural products and may be explained by several factors. First, the net effect in vivo is the sum of complex systemic, hormonal, and metabolic feedback not present in a purified enzyme assay. Second, dose-dependency is a hallmark of phytopharmaceuticals; low systemic doses might have a trophic or signaling effect, while the higher concentrations used here for direct enzyme assays are inhibitory. Finally, crude extracts contain hundreds of compounds, and it is possible that other constituents have opposing, upregulating effects that dominate in a whole-organism context. The present study clarifies that the tanshinone components are, in fact, direct inhibitors.

The cytotoxicity exhibited by all tested compounds in the low micromolar range presents a dual consideration. In the context of prostate cancer, where tanshinones have been studied for their anti-proliferative effects, this cytotoxicity could be a therapeutically beneficial feature, contributing to tumor cell killing via mechanisms independent of androgen suppression [52,71,72,73,74,75,76,77,78,79,80,81,82]. These compounds could serve as alternatives or adjuncts to existing therapies, maintaining effective androgen suppression while minimizing endocrine side effects [52,74]. However, for chronic, non-lethal conditions like congenital adrenal hyperplasia and PCOS, this cytotoxicity defines the therapeutic index. Moreover, as excessive androgen production in congenital adrenal hyperplasia and PCOS worsens metabolic and reproductive dysfunction, selective lyase inhibition could normalize androgen levels without impairing cortisol or aldosterone synthesis, thereby improving patient outcomes and adherence [83,84]. The ideal drug would have a wide margin between the effective concentration for enzyme inhibition and the concentration that induces cell death. While DT emerges as a highly promising lead, our comprehensive preclinical assessment highlights that the tanshinone scaffold is a double-edged sword [73,85,86,87]. The very chemical features that may contribute to its bioactivity, namely the reactive quinone moiety, are also flagged by PAINS and Brenk alerts and are likely contributors to the observed cytotoxicity and the predicted liabilities of broad-spectrum CYP inhibition and immunotoxicity (Table 3 and Table 4). However, use of SM in traditional medicine has a long and well tolerated safety profile and DT itself has been extensively studied [40,41,42,43,44,47,48,49,88,89,90,91,92,93,94,95,96,97,98]. These findings are not a disqualification but rather a critical roadmap for the next stage of development. They define the essential task for a future medicinal chemistry campaign: to systematically modify the tanshinone core to disentangle the desired on-target pharmacology from the off-target liabilities. This could involve, for example, isosteric replacement of the quinone system to reduce reactivity while preserving the geometry required for selective interaction with the CYP17A1 active site [99].

## 5. Conclusions

We have identified and characterized dihydrotanshinone as a potent and selective natural product-based inhibitor of CYP17A1 17,20-lyase. Its ability to suppress androgen synthesis while sparing both the critical 17α-hydroxylase function and the off-target enzyme CYP21A2 offers a significant conceptual advantage over existing non-selective inhibitors. Our integrated experimental and computational approach resolved a key structure–activity paradox, generating the hypothesis that dihydrotanshinone’s unique binding orientation, rather than its absolute binding affinity, is the primary determinant of its superior and selective function. Dihydrotanshinone therefore stands as a validated lead scaffold, providing a robust foundation for a targeted medicinal chemistry program aimed at developing a new generation of safer, more effective therapeutics for the treatment of PCOS, congenital adrenal hyperplasia, and other disorders of androgen excess.

## Figures and Tables

**Table 2:** Cytotoxicity (IC_50_) of SM extract and purified tanshinones in human cell lines. Values are presented as mean ± standard deviation from three independent experiments.

Drug	NCI-H295R (IC_50_)	HEK-293T (IC_50_)	RWPE-1 (IC_50_)
SM	2.11 ± 1.80 μg/mL	4.17 ± 2.56 μg/mL	1.12 ± 0.31 μg/mL
TAI	1.13 ± 0.63 μM	1.08 ± 0.26 μM	2.27 ± 3.25 μM
TAII	1.20 ± 3.44 μM	2.26 ± 1.92 μM	4.50 ± 11.80 μM
DT	0.95 ± 0.48 μM	1.31 ± 0.47 μM	1.40 ± 0.42 μM

**Table 3:** Comparative in Silico ADME and physicochemical profile of tanshinones from SwissADME predictions.

Parameter	Tanshinone I (TAI)	Tanshinone IIA (TAII)	Dihydrotanshinone I (DT)
Formula	C_18_H_12_O_3_	C_19_H_18_O_3_	C_18_H_14_O_3_
MW (g/mol)	276.29	294.34	278.30
Consensus Log P	3.40	3.80	3.03
TPSA (Å^2^)	47.28	47.28	43.37
ESOL Solubility Class	Moderately soluble	Moderately soluble	Soluble
GI Absorption	High	High	High
BBB Permeant	Yes	Yes	Yes
P-gp Substrate	No	Yes	No
CYP1A2 Inhibitor	Yes	Yes	Yes
CYP2C19 Inhibitor	Yes	Yes	Yes
CYP2C9 Inhibitor	No	Yes	Yes
CYP2D6 Inhibitor	No	Yes	Yes
CYP3A4 Inhibitor	Yes	Yes	Yes
Bioavailability Score	0.55	0.55	0.85
Lipinski Violations	0	0	0
PAINS Alerts	2	2	2

**Table 4:** Summary of key in silico toxicity predictions for tanshinones from ProTox-3.0 predictions. “Active” predictions are highlighted in bold.

Toxicity Endpoint	Tanshinone I (TAI)	Tanshinone IIA (TAII)	Dihydrotanshinone I (DT)
**Hepatotoxicity**	Inactive (0.63)	Inactive (0.71)	Inactive (0.62)
**Respiratory Toxicity**	**Active (0.63)**	**Active (0.56)**	**Active (0.66)**
**Carcinogenicity**	Inactive (0.51)	Inactive (0.56)	**Active (0.50)**
**Immunotoxicity**	Inactive (0.66)	Inactive (0.80)	**Active (0.98)**
**Mutagenicity**	Inactive (0.55)	Inactive (0.70)	Inactive (0.55)
**GABA Receptor Activity**	**Active (0.51)**	**Active (0.59)**	**Active (0.50)**
**AChE Inhibition**	Inactive (0.87)	Inactive (0.77)	**Active (0.79)**
**PXR Activation**	Inactive (0.59)	**Active (0.58)**	**Active (0.58)**

**Table 5:** Summary of enzyme inhibition and calculated selectivity indices. Data are derived from Figure 4 and Figure 5. Inhibition percentages calculated as (100%—% Residual Activity). ^a^ Calculated as (% Inhibition Lyase)/(% Inhibition OH). ^b^ Calculated as (% Inhibition Lyase)/(% Inhibition CYP21A2). ^c^ Selectivity index is mathematically high but therapeutically limited due to low absolute potency against the lyase activity at the tested concentration.

Compound	% Inhibition CYP17A1-Lyase	% Inhibition CYP17A1-OH	% Inhibition CYP21A2	Lyase/OH Selectivity Index ^a^	Lyase/CYP21A2 Selectivity Index ^b^
SM	39.2%	5.6%	5.0%	6.96	7.82
TAI	28.2%	<1%	<1%	High ^c^	High ^c^
TAII	43.0%	<1%	<1%	High ^c^	High ^c^
DT	56.6%	6.5%	14.9%	**8** **.67**	**3.80**
Abiraterone	69.6%	94.7%	29.8%	**0.73**	**2.34**

## Data Availability

The data that support the findings of this study are openly available Harvard Dataverse at https://doi.org/10.7910/DVN/YMX0MN (accessed on 1 January 2026) [100].

## Supplementary Materials

The following supporting information can be downloaded at https://www.mdpi.com/article/10.3390/biom16010144/s1, Figure S1: Original Western blot images.
