# Supplementary material for: Dihydrotanshinone as a Natural Product-Based CYP17A1 Lyase Inhibitor for Hyperandrogenic Disorders
Source: Biomolecules. 2026 Jan 14;16(1):144. doi: 10.3390/biom16010144 (PMC12838644; doi:10.3390/biom16010144)
Supplement: Supplementary file 1 [file biomolecules-16-00144-s001.zip › biomolecules-4051036-supplementary.pdf]

Supplementary Figure S1. Original western blot image of CYP21A2 quantification.

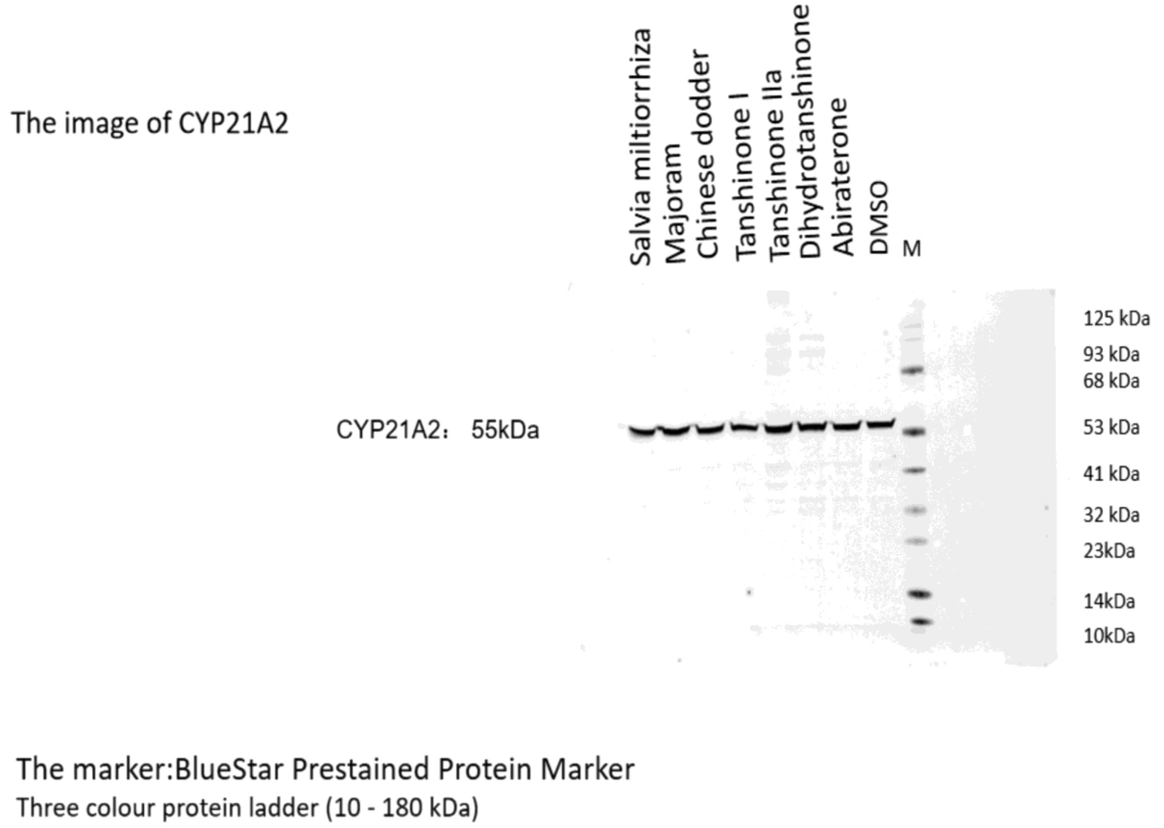

Original western Blot image for CYP21A2. The original western blot images also include two extra compounds Marjoram and Chinese dodder, which are not part of the current study and were loaded as part of a larger set.

The image of β-actin

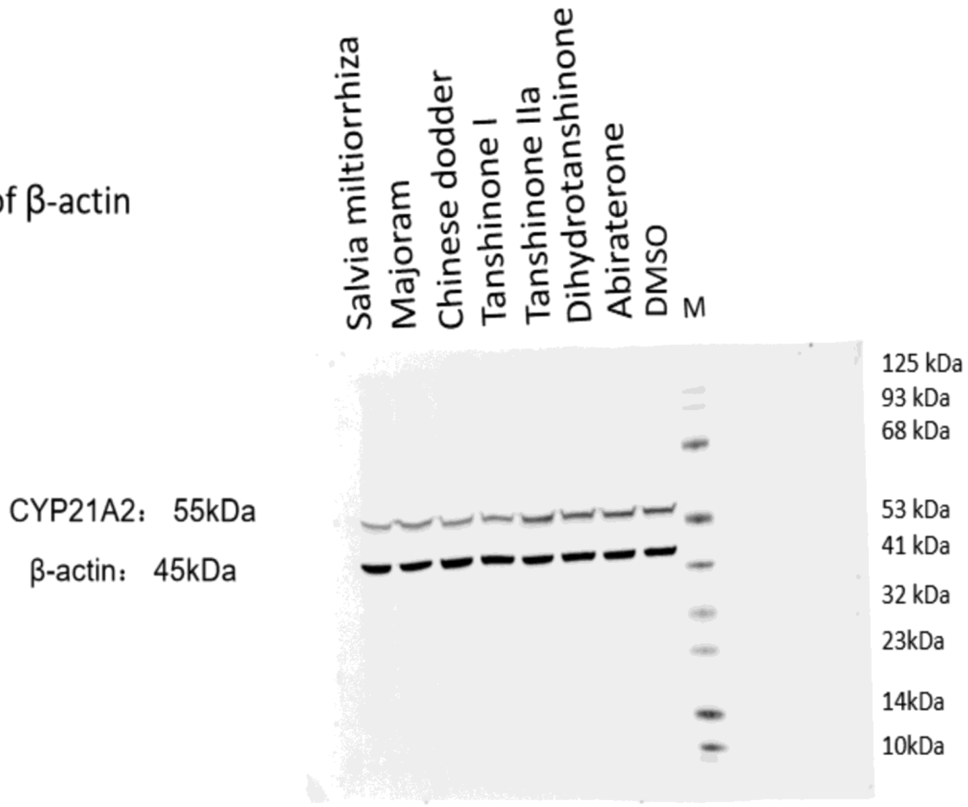

The marker:BlueStar Prestained Protein Marker

Three colour protein ladder (10 - 180 kDa)

Original Western Blot image of Beta-Actin, control antibody. The original western blot images also include two extra compounds Marjoram and Chinese dodder, which are not part of the current study and were loaded as part of a larger set.
